# Cardiac manifestations in a Chinese cohort of fetuses from mothers with anti-Ro and anti-La antibodies

**DOI:** 10.3389/fped.2022.904138

**Published:** 2022-07-28

**Authors:** Xin Wang, Xiao-wei Liu, Ling Han, Meng-tao Li, Jiu-liang Zhao, Lin Sun, Jian-cheng Han, Xiao-feng Zeng, Xin-ping Tian, Ying Zhao, Yi-hua He

**Affiliations:** ^1^Echocardiography Medical Center, Maternal-Fetal Medicine Center in Fetal Heart Disease, Beijing Anzhen Hospital, Capital Medical University, Beijing, China; ^2^Department of Pediatric Cardiology, Beijing Anzhen Hospital, Capital Medical University, Beijing, China; ^3^Department of Rheumatology, Peking Union Medical College Hospital, Chinese Academy of Medical Sciences, National Clinical Research Center for Dermatologic and Immunologic Diseases, Key Laboratory of Rheumatology and Clinical Immunology, Ministry of Education, Beijing, China

**Keywords:** endocardial fibroelastosis, anti-Ro antibodies, atrioventricular block, prenatal diagnosis, echocardiography

## Abstract

**Objectives:**

To analyze the clinical characteristics, echocardiographic features, and prognosis of fetuses based on three groups of cardiac manifestations associated with maternal anti-Ro and anti-La antibodies in China. This study included three groups: the isolated-arrhythmia, isolated-endocardial fibroelastosis (EFE), and mixed groups.

**Methods:**

We prospectively evaluated 36 fetuses with cardiac manifestations due to maternal anti-Ro and anti-La antibodies from our center between 2016 and 2020 in China. Clinical and echocardiographic data were collected.

**Results:**

There were 13 patients (36%) in the isolated-arrhythmia group, eight (22%) in the isolated-EFE group, and 15 (42%) in the mixed group. All patients in the isolated-EFE group presented with mild EFE. Severe EFE was identified in four patients (27%) in the mixed group. Atrioventricular block (AVB) was more common in the isolated-arrhythmia group (13, 100%) than in the mixed group (6, 40%; *p* = 0.001). Moderate-severe mitral regurgitation (*p* = 0.006), dilated cardiomyopathy (DCM, *p* = 0.017), and low cardiovascular profile scores (*p* = 0.013) were more common in the mixed group than in the other two groups. Twenty-one mothers decided to terminate the pregnancy and 15 fetuses were born with regular perinatal treatment. They all survived at 1 year of age. One patient in the isolated-arrhythmia group and two in the mixed group required a pacemaker due to third-degree AVB or atrioventricular junctional rhythm. Five patients in the isolated-EFE group and five in the mixed group had no DCM or heart failure and the location of mild EFE was significantly reduced.

**Conclusion:**

Fetal cardiac manifestations due to maternal anti-Ro and anti-La antibodies can be divided into three groups, i.e., the isolated-arrhythmia, isolated-EFE, and mixed groups. AVB usually occurs in the isolated-arrhythmia group. Severe EFE, moderate-severe mitral regurgitation, and DCM mainly appear in the mixed group. Location of mild EFE significantly reduces after birth and the outcome of fetuses with mild EFE depends on the presence of arrhythmia and its subtypes.

## Introduction

The placental transference of maternal anti-Ro and anti-La antibodies is associated with neonatal lupus erythematosus syndrome (NLES), in which there are various cardiac complications (1–3). Arrhythmia is the most common cardiac manifestation in this condition, especially congenital heart block (CHB). There are three types of CHB and third-degree atrioventricular block (AVB) is permanent and usually requires pacemaker implantation. Other cardiac manifestations are structural or functional abnormalities, containing endocardial fibroelastosis (EFE), valvular disease, and late-onset dilated cardiomyopathy (DCM) (4–6).

According to previous reports, cardiac manifestations of NLES can be divided into three types, including the isolated-arrhythmia, isolated-EFE, and mixed (arrhythmia with EFE) types (4–8). There has been research summarizing the clinical and ultrasonic features of NLES. About 93% of babies with CHB have no EFE and their outcome mainly depends on the subtypes of CHB and the ventricular rate. Guettrot-Imbert et al. (10) identified five patients with isolated EFE and their middle-term prognosis was better than previous reports. Nield et al. (9) collected 13 children in the mixed group (third-degree AVB with severe EFE) with nine deaths and two cardiac transplantations. However, there has been no large-scale study describing the clinical features and prognosis of the three cardiac types of fetuses due to maternal anti-Ro and anti-La antibodies.

In this report, our primary objective was to summarize clinical features of 36 Chinese fetuses with heart damage due to maternal anti-Ro and anti-La antibodies and their outcome. Our secondary objective was to demonstrate the incidence and outcome of fetuses with EFE in this condition.

## Materials and methods

### Patient population

Our is a prenatal diagnosis center for fetal cardiac abnormalities, receiving fetuses from all over China. From 2016 to 2020, we prospectively collected 36 consecutive fetuses with cardiac manifestations due to maternal anti-Ro and anti-La antibodies. The institutional research ethics board of Beijing Anzhen Hospital approved this prospective observational study. All pregnant women signed written informed consent prior to their inclusion into this study.

### Fetal echocardiography

Two- and three-dimensional ultrasound systems Voluson E8 and Voluson E10 (GE Healthcare, Zipf, Austria) with 2–8 MHz transabdominal curved array probes were adopted in this work. The prolongation of the A-V interval (>150 ms) was considered first-degree AVB. Second-degree AVB showed the A-V block of one or more, but not all. Type 1 second-degree AVB manifested a progressive prolongation of the A-V interval until one A wave failed to conduct to the ventricle. Intermittent non-conducted A waves without prolongation of the A-V interval were defined as type 2 second-degree AVB. The absence of A-V conduction was defined as third-degree AVB. EFE was defined as bright and white locations of endocardial echogenicity, with defined margins. Sites of mild EFE mainly enriched the surface of the atrial wall, atrioventricular valve, and atrial septum or semilunar valve. Sites of severe EFE should include the surface of the left or right ventricles. DCM was identified as left or right ventricular enlargement with a shortening fraction <28%. The cardiovascular profile scores (CVPS) were used to assess fetal heart failure and prognosis (11).

### Data collection and follow-up

Clinical data, including family history, maternal presence of anti-Ro or anti-La antibodies, antenatal therapy, pregnancy outcome and postnatal therapy, were collected. After cardiac manifestations were found, the pregnant women were referred to the Rheumatology Unit for standard treatment and regular echocardiographic examinations. The perinatal outcome was followed up in the outpatient clinic. Data of the echocardiography and electrocardiogram were recorded at 1 month and 1 year, when possible.

### Statistical analysis

We used IBM SPSS Statistics for Windows (version 25.0, Armonk, NY, USA) to analyze the data. Medians with ranges and means with standard deviations were calculated whenever appropriate. The statistical significance of the differences between the means of the two groups was assessed using unpaired *Student's t–test*. The *Kruskal–Wallis H test*, χ^2^ *test* and *Fisher's exact test* were used to compare the clinical and echocardiographic features for statistical analysis. All tests were two-sided, and statistical significance was set at *p* < 0.05.

## Results

### General information

**Figure 1:**
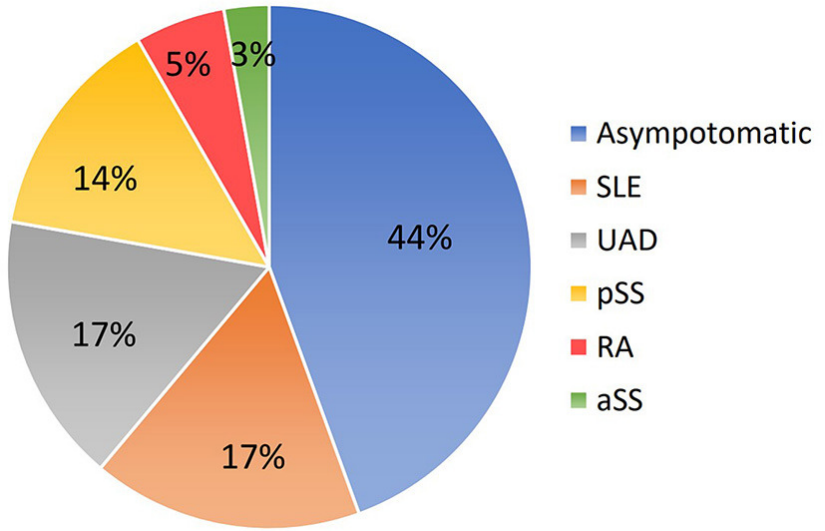
Conditions related to mothers carrying anti-Ro and anti-La antibodies. SLE, systemic lupus erythematosus; UAD, undifferentiated autoimmune disease; pSS, primary Sjögren syndrome; RA, rheumatoid arthritis; aSS, Sjögren syndrome associated with other systemic autoimmune diseases.

The mean maternal age was 30.2 ± 4.3 years (range 20–40 years). The average gestational age at diagnosis with cardiac manifestations was 25.0 ± 3.5 weeks (range 20–32 weeks). Nineteen cardiac-affected mothers were positive for anti-Ro and anti-La antibodies, and 17 were positive for anti-Ro antibody only. Antibodies anti-Ro52 and anti-Ro60 were positive in 100% and 81% (29/36) of the patients respectively. About 44% (16/36) of mothers were asymptomatic Ro/La carriers, while 56% (20/36) of them were affected by an autoimmune disease (Figure 1). Maternal autoimmune diseases before echocardiographic examination were confirmed in 11 patients. Those pregnant women took regular treatment in perinatal periods. Twenty-three women were discovered to be Ro/La carriers only after the diagnosis of cardiac disease in their fetuses. Those pregnant women took medical treatment after echocardiographic examination if they continued with the pregnancy.

### Echocardiographic data

**Figure 2:**
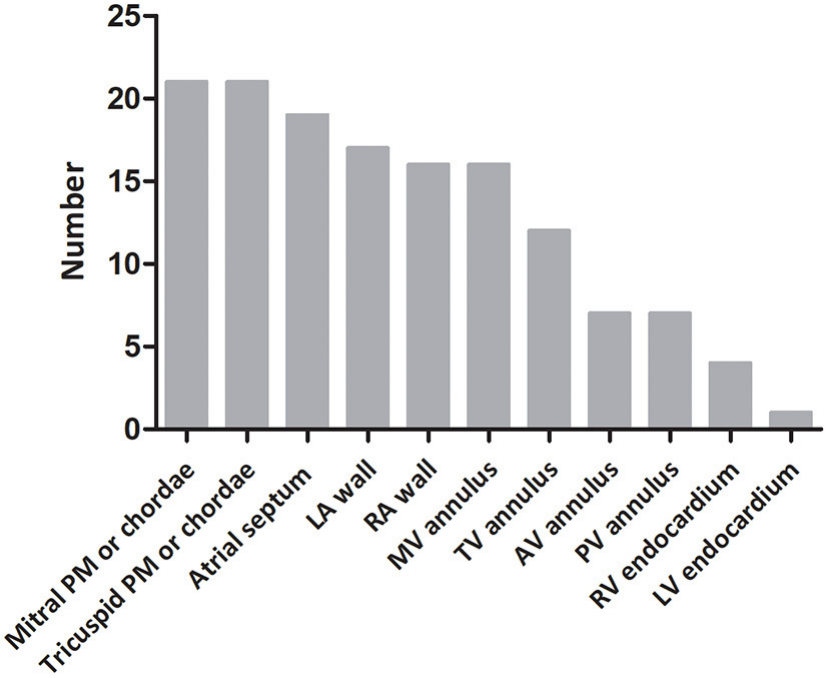
Distribution of endocardial fibroelastosis in fetuses with cardiac complications due to maternal anti-Ro and anti-La antibodies. PM, papillary muscle; LA, left atrium; RA, right atrium; MV, mitral valve; TV, tricuspid valve; AV, aortic valve; PV, pulmonary valve; RV, right ventricle; LV, left ventricle.

**Figure 3:**
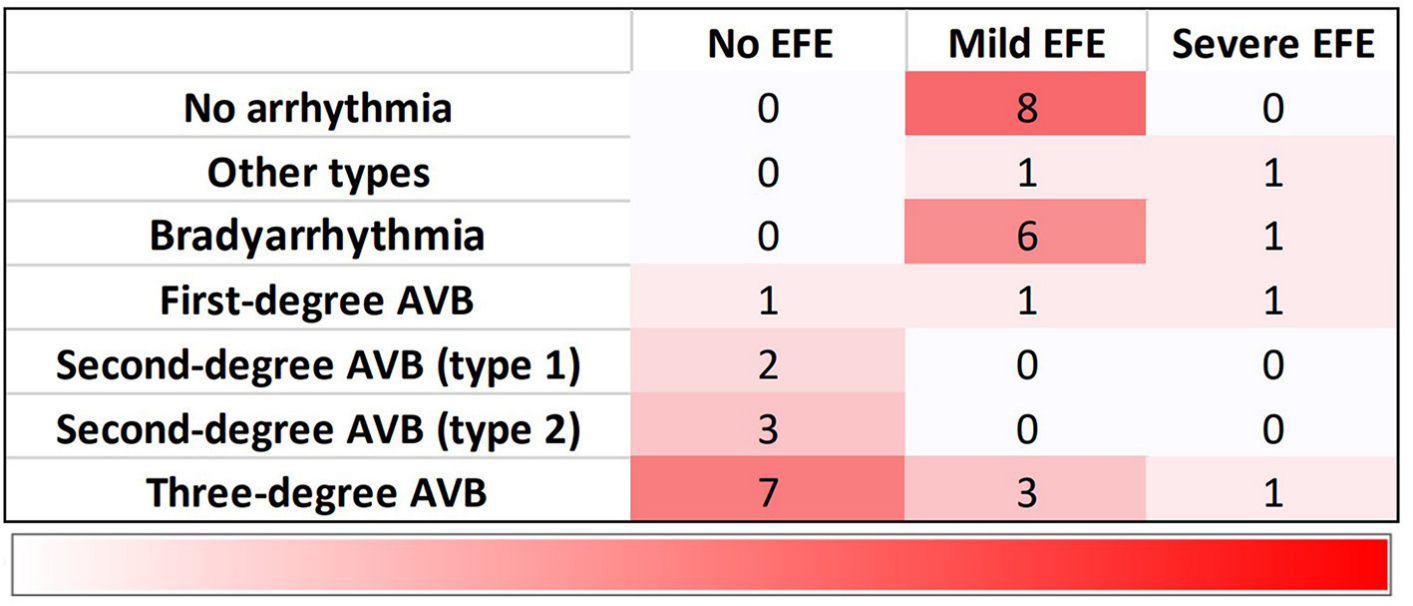
Distribution of 36 fetuses with cardiac manifestations due to maternal anti-Ro and anti-La antibodies. Bradyarrhythmia includes sinus bradycardia, junctional rhythm and ectopic atrial rhythm. Other types contain ventricular tachycardia and atrial flutter. EFE, endocardial fibroelastosis; AVB, atrioventricular block.

Arrhythmias were detected in 78% of fetuses (28/36), EFE in 64% (23/36). CHB was the most common cardiac manifestation (53%, 19/36) and 31% (11/36) of fetuses had third-degree AVB. Bradyarrhythmia with a 1:1 AV relationship and normal mechanical PR interval was observed in 19% of patients (7/36). Bradyarrhythmia *in utero* included sinus bradycardia, junctional rhythm and ectopic atrial rhythm. Ventricular tachycardia and persistent atrial flutter could be found in two fetuses. EFE was hyperechogenic of the affected area, mainly on the mitral and tricuspid papillary muscles or chordae (91%, 21/23), atrial septum (83%, 19/23), and left atrial wall (74%, 17/23; Figure 2). The atrioventricular annulus, semilunar annulus, and the endocardial regions of ventricles were also hyperechogenic. Figure 3 shows the various combinations of arrhythmias and EFE in our study.

According to data of fetal echocardiography, all patients were divided into three groups, containing isolated-arrhythmia group (13/36 patients, 36%), isolated-EFE group (8/36 patients, 22%), and mixed group (15/36 patients, 42%). Table 1 shows the clinical and echocardiographic data of the three groups. CHB was more common in the isolated-arrhythmia group (100%) than in the mixed group (40%, *p* = 0.001). All patients with moderate-severe mitral regurgitation (*p* = 0.006) and DCM (*p* = 0.017) were in the mixed group. Patients in the mixed group had the lowest CVPS (*p* = 0.013). All patients in the isolated-EFE group presented with mild EFE. Out of the 15 patients in the mixed group, mild and severe EFE was seen in 11 and four patients, respectively.

**Table 1:** Clinical and echocardiographic data of fetuses with three cardiac manifestations due to maternal anti-Ro and anti-La antibodies.

	**Isolated arrhythmia (*n* = 13)**	**Isolated EFE (*n* = 8)**	**Mixed (*n* = 15)**	***p*-Value**
Maternal age (years)	30.2 ± 4.9	32.6 ± 2.9	28.8 ± 4.3	0.131
Anti-Ro(+) and anti-La(+)	6 (46)	3 (38)	10 (67)	0.111
Anti-Ro(+) alone	7 (54)	5 (62)	5 (33)	
Positive anti-Ro52 Ab	13 (100)	8 (100)	15 (100)	1.000
Positive anti-Ro60 Ab	11 (85)	7 (88)	13 (87)	0.980
Gestational age (weeks)	26.0 ± 3.8	24.2 ± 3.9	25.0 ± 3.5	0.444
Mild EFE	/	8	11	0.257
Severe EFE	/	0	4	
CHB	13	/	6	0.001
Other arrhythmias	0	/	9	
Moderate-severe MR	0	0	6	0.006
Moderate-severe TR	2	1	7	0.101
DCM	0	0	5	0.017
CVPS (scores)	8.7 ± 1.3	9.5 ± 0.8	7.7 ± 1.7	0.013
Pericardial effusion	0	1	5	0.058
Hydrops	1	0	2	0.529

EFE, endocardial fibroelastosis; CHB, congenital heart block; MR, mitral regurgitation; TR, tricuspid regurgitation; DCM, dilated cardiomyopathy; CVPS, cardiovascular profile scores.

### Follow-up

Sixteen mothers decided to interrupt their pregnancy after their fetuses were diagnosed with cardiac damage. Five others received medical treatment for 2–4 weeks, however, as no clinical response was observed at the fetal echocardiography, they chose to terminate their pregnancy.

Fifteen children were born: 40% (6/15) were premature, 47% (7/15) presented a low birth weight, and 33% (5/15) an intrauterine growth restriction. Eight pregnant women were diagnosed with an autoimmune disease before their pregnancy. All of them have been treated with dexamethasone (4 mg/day), six also received hydroxychloroquine (400 mg/day) during the first trimester, and another one intravenous immunoglobulins (1 g/day) during the second trimester, once EFE was diagnosed in her fetus. Seven asymptomatic carriers of anti-Ro and anti-La antibodies had been identified after the diagnoses of cardiac involvement in their fetuses, and had been treated with dexamethasone (4 mg/day) in the second trimester. Three women received intravenous immunoglobulins (1 g/day) when their fetuses have been diagnosed with EFE, in the second trimester.

**Figure 4:**
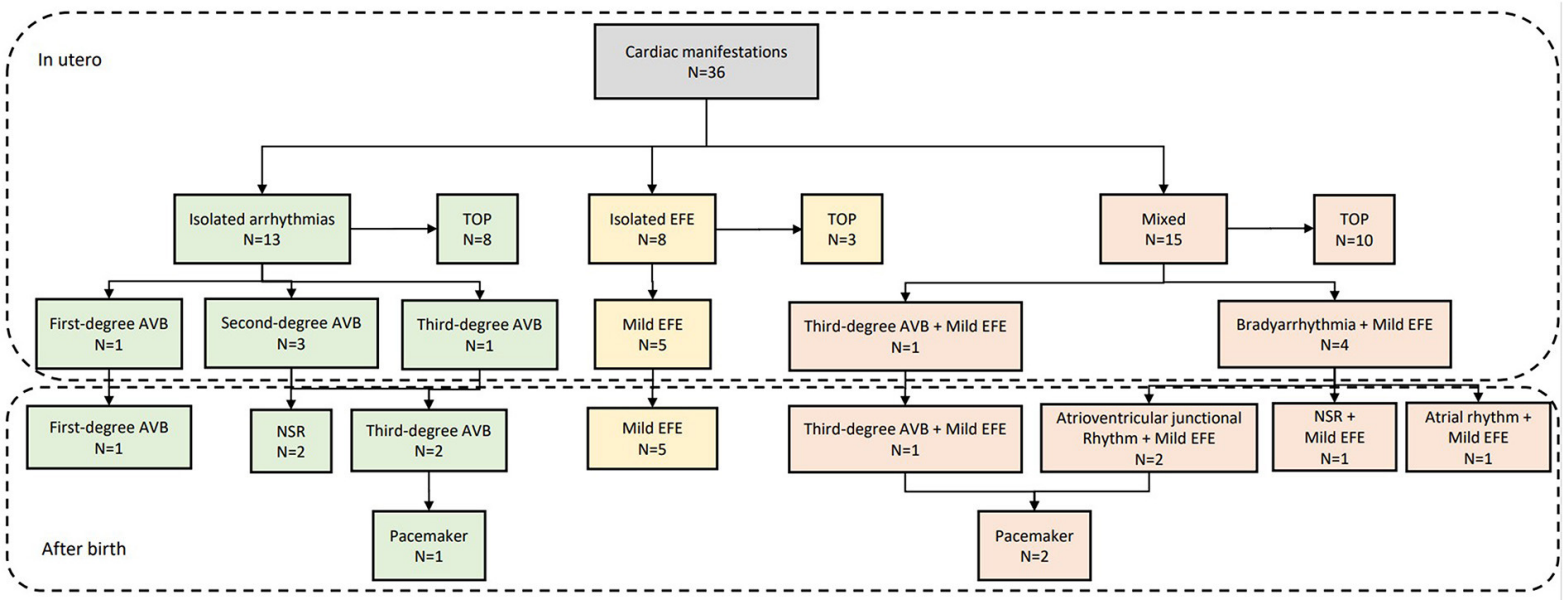
Outcome of fetuses with cardiac manifestations due to maternal anti-Ro and anti-La antibodies. TOP, termination of pregnancy; EFE, endocardial fibroelastosis; AVB, atrioventricular block; NSR, normal sinus rhythm.

**Figure 5:**
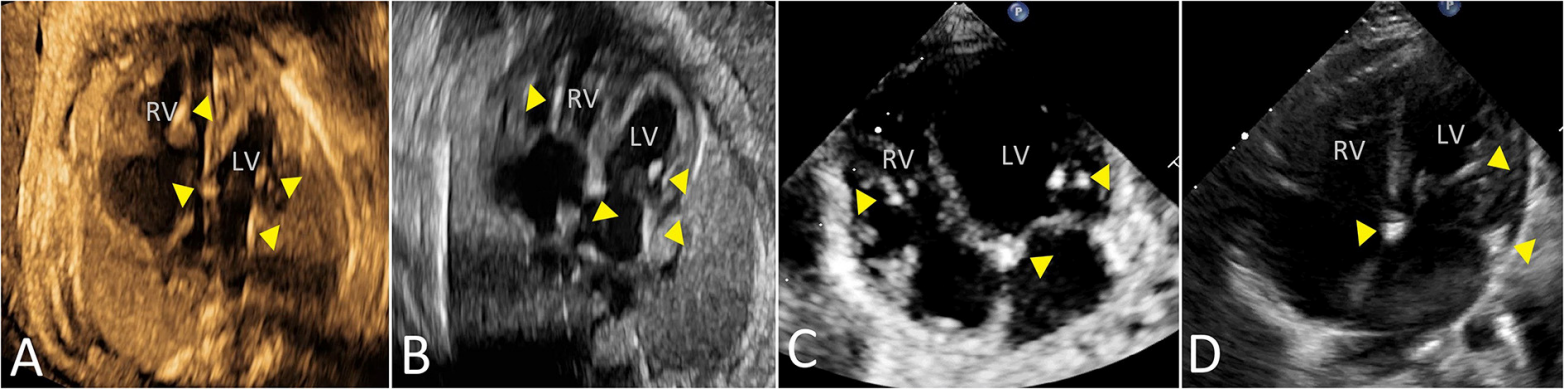
The echocardiographic features of isolated mild EFE in a fetus due to maternal anti-Ro and anti-La antibodies. Triangles mark the areas of mild EFE in four-chamber view [**(A)** 30 gestational weeks; **(B)** 33 gestational weeks; **(C)** 1 month; **(D)** 1 year]. LV, left ventricle; RV, right ventricle.

Figure 4 shows data related to 1-year outcome of newborn infants. One child with first-degree AVB *in utero* was diagnosed with first-degree AVB after birth without heart dysfunction or DCM at 1 year of age. Two children with type 1 second-degree AVB *in utero* were diagnosed with normal sinus rhythm after birth. One child with type 2 second-degree AVB *in utero* developed third-degree AVB after birth. One child, presenting with third-degree AVB during the prenatal and postnatal periods, was fitted with a pacemaker at 5 months of age. Five infants, who had mild EFE in the fetal period, showed normal atrioventricular conduction with mild EFE after birth. However, echocardiography at 1 year old showed that the regions of EFE were reduced or even disappeared in five patients. The main areas of hyperechogenicity by echocardiography were observed in the mitral and tricuspid papillary muscles or chordae and atrial septum. One child had a pacemaker at 7 months old because she had third-degree AVB with mild EFE before and after birth. Four children with bradyarrhythmia (atrioventricular conduction ratio = 1:1) and mild EFE in fetal periods presented with different manifestations after birth. Two patients presented with atrioventricular junctional rhythm, one with normal sinus rhythm and the other with atrial rhythm. Mild EFE did not disappear, but areas of EFE markedly shrunk. The duration of follow-up was from 1 to 5 years. There was no heart failure or late-onset DCM observed in our study during follow-up. Figure 5 shows the echocardiographic data in the perinatal period of one case with isolated EFE.

Other extracardial manifestations of NLES could be found at birth. Cutaneous lesions were observed in 11 patients and presented with annular erythema in sun-exposed areas. Four patients were thrombocytopenic. Only two patients showed elevation of alanine transaminase and aspartate transaminase. There was no neurological abnormality observed in our study.

## Discussion

Maternal anti-Ro and anti-La antibodies can result in NLES, which is characterized by a spectrum of diseases of the heart, liver, and skin in fetuses or newborns. Cardiac manifestations are not rare and third-degree AVB is usually considered an irreversible disorder (3, 12–14). It is the most common cardiac manifestation, which is isolated or associated with EFE (9, 12, 14). EFE can be a severe consequence of fetal myocarditis, which can appear in the absence of CHB (10, 14). Previous reports described three types of cardiac manifestations separately. However, there is no systematic description of the three cardiac types. Therefore, we summarized clinical features and outcomes of the three cardiac types in 36 fetuses with cardiac manifestations due to maternal anti-Ro and anti-La antibodies in China. The mixed manifestation, manifesting arrhythmia with EFE, was the most common in this study and severe EFE was only identified in the mixed type. CHB usually existed in isolation. Fetuses with isolated mild EFE had good prognosis in the short term.

Only a part of seropositive mothers is aware of their anti-Ro and anti-La antibodies positivity, as they have a diagnosis of connectivitis. Previous systematic reviews found that 53% of mothers with CHB babies were asymptomatic carriers without specific autoimmune diseases (2, 12, 15–17). Undifferentiated autoimmune disease, primary Sjögren syndrome and systemic lupus erythematosus were common in this condition. In our study, of the 36 affected mothers, nearly half were classified as asymptomatic carriers of these two antibodies, while about 17% had undifferentiated autoimmune disease and 17% had systemic lupus erythematosus.

All mothers in our study had positive anti-Ro antibodies, and 47% were positive for both anti-Ro and anti-La antibodies. There was no case of cardiac manifestations associated with only anti-La antibody positivity in our study, which was consistent with previous reports (12, 18, 19). Therefore, monitoring asymptomatic mothers with anti-Ro or anti-La antibodies and their fetuses due to potential development of autoimmune diseases can ensure the timely and in-depth treatment to protect their wellbeing. The distributions of arrhythmias and associated cardiac abnormalities in this study are slightly different from those reported previously. AVB is the most common fetal cardiac manifestation in fetuses related with maternal anti-Ro or anti-La antibodies (2–6, 12–15). Third-degree AVB is the most severe form with an incidence of >80% in literature (20), and EFE is less common, observed in 7% of babies affected by CHB (12, 21). In our study, CHB was identified in 53% of patients, and 31% had third-degree AVB. EFE could be detected in 32% (6/19) of fetuses affected with CHB. This difference may be associated with an ethnic variation, close perinatal management, and extensive clinical experience with this condition. Other electrophysiological abnormalities have been detected in fetuses with maternal antibodies, including sinus node dysfunction, atrial flutter, ventricular and junctional tachycardia, and long QT interval (22–26). About 19% of fetuses in our study had sinus bradycardia with mild or severe EFE, which had a higher occurrence than previous reports. Postnatal progression of sinus bradycardia is possible, developing into normal rhythm or sinus bradycardia as described in previous research (27) and in our study. The histopathology revealed extensive fibrosis and calcification of the sinus node, atrioventricular node, and even the entire conduction system (27).

Third-degree AVB is irreversible, and several studies found no potential positive contribution of antenatal steroid therapy to improve the prognosis of these fetuses (28–30). Although fluorinated steroids are usually used in fetuses with second-degree AVB to prevent *in utero* progression, there is no evidence for the benefit of antenatal steroid therapy affecting the outcome of fetuses with second-degree AVB (31). Hydroxychloroquine is prophylactic and considered to reduce the incidence of AVB in fetuses with maternal antibodies. It is usually used in combination with glucocorticoid. Fetal sinus bradycardia may carry good outcomes in the absence of EFE or cardiomyopathy. The pacemaker is implanted for progressive sinus node dysfunction after long-term follow-up.

Endocardial fibroelastosis is a rare abnormality, which can involve endocardium of ventricles and lead to heart failure or neonatal death (9, 32). The association between EFE and autoantibody-related CHB was firstly described by Hogg in 1957 (33). Severe EFE, with predominant involvement of the ventricle, is usually combined with third-degree AVB and has severe ventricular dysfunction, cardiac death, or requires cardiac transplantation (33). In contrast, mild EFE, with predominant involvement of atrial walls, atrioventricular valve, papillary muscles, or chordae, can exist in isolation and has good outcomes (10, 34). Our study found four patients with severe EFE with arrhythmias. Two pregnancies were terminated due to the finding of DCM, pericardial effusion, and atrioventricular regurgitation, although the mothers had been treated with intravenous immunoglobulins and dexamethasone for 4 weeks. However, no signs of improvement were observed in this period. All patients with mild EFE had no DCM or cardiac dysfunction during the follow-up, and they did not require any medical treatment. Furthermore, the involved areas predominantly decreased or even disappeared. We found EFE to be not rare and its treatment remains controversial. Fluorinated steroids have been used in fetuses with isolated EFE, but their efficacy is unclear (10). Side-effects include intrauterine growth restriction, hydrops, and stillbirth (35). All eight fetuses with isolated EFE in our study took dexamethasone and two of them had intrauterine growth restriction. We did not observe hydrops, stillbirth, or DCM. However, this result could not confirm the superiorly therapeutic effect of fluorinated steroids for isolated EFE because its long-term prognosis was not as observed in our study and other reports (10).

This study has some limitations. Although our data have been collected in a referral center for fetal echocardiography, the small size of our sample is one of the limitations of this study. Given only 15 fetuses with intact history, this study was unable to predict pregnancy outcome by fetal echocardiography. Another limitation of our study was the absence of histological examinations or other useful imaging techniques to confirm the real severity of EFE. All survived patients need long-term follow-up to confirm whether they have the risk of late-onset cardiomyopathy or DCM.

In conclusion, we described the clinical and echocardiographic features of 36 Chinese fetuses with arrhythmia or EFE due to maternal anti-Ro and anti-La antibodies. Mixed presentation (arrhythmia with EFE) resulted to be the most common manifestation. Severe EFE was identified only in the mixed type. CHB usually existed as a single manifestation. Isolated mild EFE had a good prognosis in the short term.

## Data availability statement

The original contributions presented in the study are included in the article/supplementary material, further inquiries can be directed to the corresponding authors.

## Ethics statement

This study protocol was reviewed and approved by the Medical Ethics Committee of Beijing Anzhen Hospital. All these pregnancy mothers or their families wrote informed consent.

## Author contributions

XW: acquisition of data, analysis and interpretation of data, and drafting of manuscript. X-pT, YZ, and Y-hH: study concept and design. X-wL, LH, M-tL, J-lZ, LS, J-cH, and X-fZ: acquisition of data. All authors were involved in the interpretation of the study results, as well as the drafting and revision of the manuscript, and approved the final version to be published.

## Funding

This work was supported by National Natural Science Foundation of China (Nos. 82170301 and 82100322), Beijing Municipal Science & Technology Commission (Z181100001918008) and Key Laboratory of Fetal Heart Disease Maternal and Child Medicine, Beijing, China (BZ0308).

## Conflict of interest

The authors declare that the research was conducted in the absence of any commercial or financial relationships that could be construed as a potential conflict of interest.

## Publisher's note

All claims expressed in this article are solely those of the authors and do not necessarily represent those of their affiliated organizations, or those of the publisher, the editors and the reviewers. Any product that may be evaluated in this article, or claim that may be made by its manufacturer, is not guaranteed or endorsed by the publisher.
